# A new species of the subgenus *Japonigekko* (Squamata, Gekkonidae, *Gekko*) from Luanchuan County, Henan Province, China

**DOI:** 10.3897/zookeys.1296.206148

**Published:** 2026-10-28

**Authors:** Yixue Zhang, Zhihao Jiang, Ruyi Huang, Song Huang, Jingjing Yang, Haoyu Wu, Zifang Ding, Wenrong Liu, Yudong Zhang, Hailong Wu

**Affiliations:** 1 Collaborative Innovation Center of Recovery and Reconstruction of Degraded Ecosystems in Wanjiang Basin Co-Founded by Anhui Province and Ministry of Education, School of Ecology and Environment, Anhui Normal University, Wuhu, China Collaborative Innovation Center of Recovery and Reconstruction of Degraded Ecosystems in Wanjiang Basin Co-Founded by Anhui Province and Ministry of Education, School of Ecology and Environment, Anhui Normal University Wuhu China; 2 The Anhui Provincial Key Laboratory of Biodiversity Conservation and Ecological Security in the Yangtze River Basin, College of Life Sciences, Anhui Normal University, Wuhu 241000, China The Anhui Provincial Key Laboratory of Biodiversity Conservation and Ecological Security in the Yangtze River Basin, College of Life Sciences, Anhui Normal University Wuhu China; 3 Shanghai Universities Key Laboratory of Marine Animal Taxonomy and Evolution, Shanghai Ocean University, Shanghai 201306, China Shanghai Universities Key Laboratory of Marine Animal Taxonomy and Evolution, Shanghai Ocean University Shanghai China

**Keywords:** *Gekko luanchuanensis* sp. nov., *
Japonigekko
*, morphology, phylogenetics, taxonomy

## Abstract

This study describes a new gecko species, *Gekko luanchuanensis***sp. nov**., from Luanchuan County, Henan Province, China. Morphologically, the new species can be distinguished from its congeners of the subgenus *Japonigekko* by the following combination of morphological characters: (1) body size moderate; (2) nostrils fused with the snout scale; (3) interorbitals 23–25; (4) tubercles small and sparse, with dorsal tubercles not forming rows at midbody; (5) midbody scale rows 141–157; (6) ventral scale rows 35–37; (7) limbs with an absence of tubercles; (8) subdigital lamellae numbers 9–11 on finger I, 10–14 on finger IV, 9–10 on toe I, and 9–13 on toe IV; (9) webbing absent; (10) precloacal pores 6–8; (11) postcloacal tubercles two on each side; (12) 6–8 dark-gray stripes on the dorsum. Phylogenetic analyses of concatenated mitochondrial 16S ribosomal RNA (16S) and Cytochrome b (Cytb) gene fragments revealed that this new species forms an independent clade with strong support, and the most closely related species is *Gekko swinhonis*, with an uncorrected *p*-distance of 13.99%. The discovery of the new species increases the number of recognized *Gekko* species worldwide to 102, the number of species worldwide in the subgenus *Japonigekko* to 40, and the number of *Japonigekko* species in China to 28.

## Introduction

The genus *Gekko* Laurenti, 1768, belongs to the family Gekkonidae (Uetz et al. 2026) and comprises seven subgenera: *Gekko* Laurenti, 1768 (nominotypical subgenus), *Japonigekko* Wood, Guo, Travers, Su, Olson, Bauer, Grismer, Siler, Moyle, Andersen & Brown, 2020, *Ptychozoon* Kuhl & van Hasselt, 1822, *Rhacogekko* Wood, Guo, Travers, Su, Olson, Bauer, Grismer, Siler, Moyle, Andersen & Brown, 2020, *Lomatodactylus* van der Hoeven, 1833, *Balawangekko* Wood, Guo, Travers, Su, Olson, Bauer, Grismer, Siler, Moyle, Andersen & Brown, 2020, and *Archipelagekko* Wood, Guo, Travers, Su, Olson, Bauer, Grismer, Siler, Moyle, Andersen & Brown, 2020 (Wood et al. 2020). The subgenus *Japonigekko* comprises 39 species, 27 of which are distributed in China (Lyu et al. 2021; Xu et al. 2025; Uetz et al. 2026). Members of the subgenus *Japonigekko* are characterized by the following diagnostic characters: body size moderate; nares typically in contact with the rostral scale; 2–4 nasals (including nasorostral, supranasal, and postnasal); 0–21 rows of dorsal tubercles; 0–32 precloacal pores; 1–4 postcloacal tubercles; and lateral folds lack tubercles (Wood et al. 2020; Hou et al. 2021; Ma et al. 2024; Wang et al. 2024; Zhou et al. 2025a; Uetz et al. 2026).

The species diversity of the subgenus *Japonigekko* has long been underestimated (Rösler et al. 2005, 2011). In the past five years, 10 new species of *Japonigekko* in China have been described (Hou et al. 2021; Lyu et al. 2021; Zhang et al. 2023; Ma et al. 2024; Wang et al. 2024; Huang et al. 2025; Xu et al. 2025; Zhou et al. 2025a, 2025b; Uetz et al. 2026; Wang et al. 2026). In particular, Hou et al. (2021) described *Gekko jinjiangensis*, and Zhou et al. (2025b) described *G. liui* as new species, based on mitochondrial NADH dehydrogenase subunit 2 (ND2) gene and morphological data. Ma et al. (2024) described *G. alpinus*, Wang et al. (2024) described *G. paucituberculatus*, Xu et al. (2025) described *G. tesselatus*, Zhou et al. (2025a) described *G. prep*, Huang et al. (2025) described *G. fengshanensis*, and Wang et al. (2026) described *G. mosaicus* as new species, based on two mitochondrial genes, 16S and ND2, and morphological data. Lyu et al. (2021) described *G. cib* as a new species based on two mitochondrial genes, 16S and Cytb, and morphological data. Zhang et al. (2023) described *G. kaiyai* as a new species, based on three mitochondrial genes, Cytb, cytochrome c oxidase subunit I (COI), and 16S, and morphological data. The species diversity and taxonomic relationships of the subgenus *Japonigekko* warrant further investigation.

*Gekko swinhonis* Günther, 1864, belongs to the subgenus *Japonigekko* and is endemic to China. The type specimen of *G. swinhonis* was collected in Pekin (Beijing), China, by R. Swinhoe (Boulenger 1885). It is widely distributed across the Loess Plateau, Huabei Plain, Huanghuai Plain, and the areas north of the Yangtze River, with its southernmost distribution reaching Zhejiang Province (Zhao et al. 1999; Yan et al. 2010). Previous studies have shown that *G. swinhonis* from different geographical units exhibit morphological differences. Zhao et al. (1999) noted that the tubercles in *G. swinhonis* from Shaanxi Province are indistinct or absent. Liang (1999) reported that specimens of *G. swinhonis* from Xi’an City exhibit significant differences in microdermatoglyphics relative to those from Xunyang City. Subsequent phylogeographic studies have shown that *G. swinhonis* is divided into two independent lineages. Yan et al. (2010) conducted a detailed study on the geographic lineage differentiation of *G. swinhonis*. Their results showed that there are two reciprocally monophyletic lineages in *G. swinhonis* (the lowland lineage and Qinling lineage), and the high genetic divergence and morphological differences suggest the presence of a cryptic species. Cao et al. (2026) conducted a detailed study on the diversity, phylogeny, and biogeography of the subgenus *Japonigekko* and reported that the *G. swinhonis* complex includes at least one lineage that may represent a distinct species.

**Figure 1.**
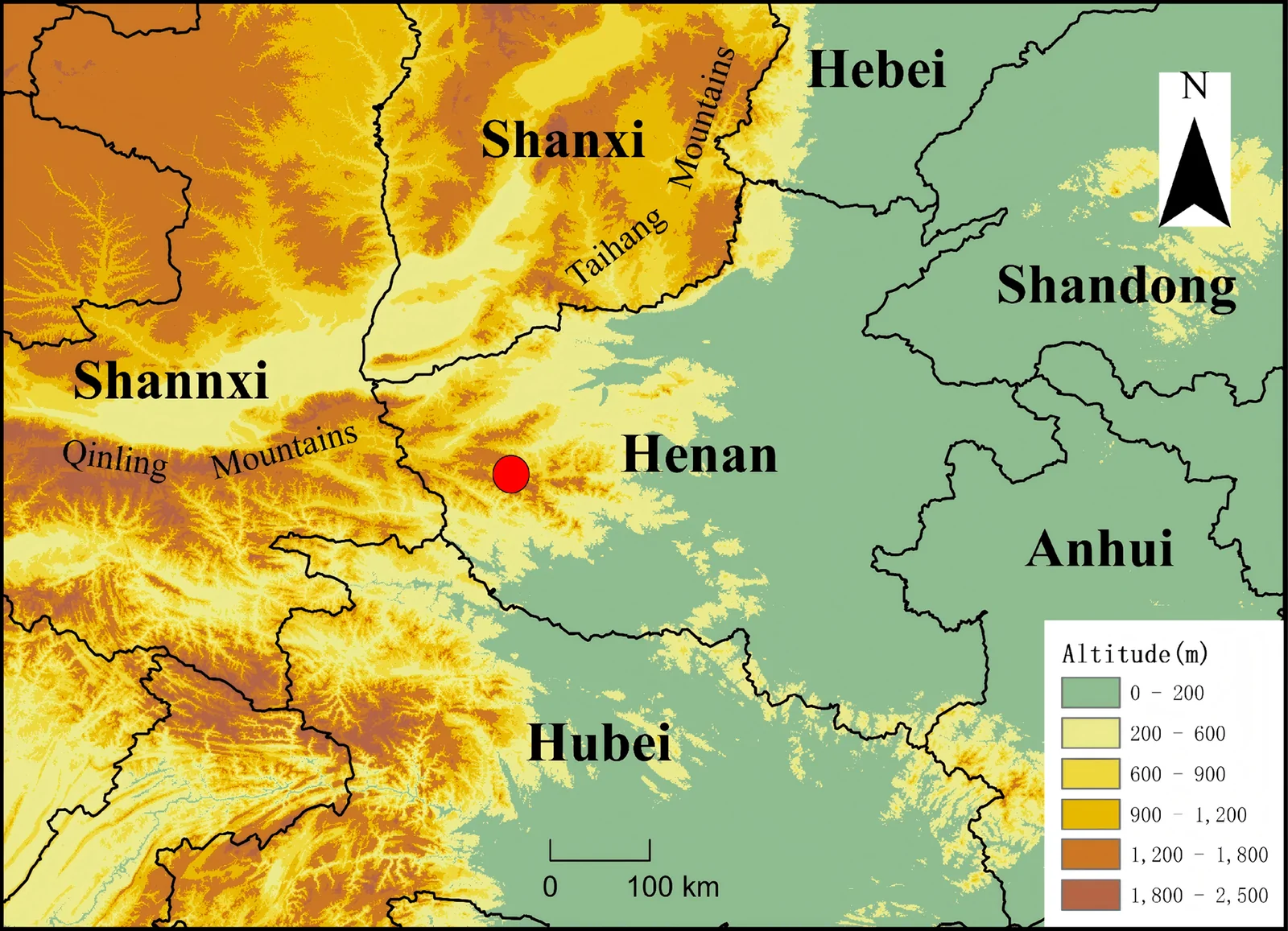
The sampling site (type locality) of *Gekko luanchuanensis* sp. nov. (red dot).

From August to September 2025, we collected three gecko samples in Luanchan County, Henan Province (Fig. 1), on the southern slope of the Qinling Mountains. Based on morphological and molecular evidence, we found that they differ from *G. swinhonis* and other members of the subgenus *Japonigekko*. We herein describe them as a new species of *Japonigekko*.

## Material and methods

### Sampling

The three specimens were collected at 33°78'N, 111°61'E, at elevations ranging from 754 to 782 m a.s.l. Their voucher numbers are HSR25010 to 12, the museum’s catalogue number are AHNU2026001 to 3. After photographing, euthanasia was performed using a 0.7% tricaine methanesulfonate (MS-222) solution (Conroy et al. 2009; Jiang et al. 2024). Liver samples were rapidly preserved in 95% ethanol for molecular analysis, while specimens were fixed in 10% formalin and stored in 75% ethanol, then kept in the museum of Anhui Normal University.

### Molecular phylogeny

Genomic DNA of each specimen was extracted from the preserved liver tissue. The primers L-14731 (Tanaka-Ueno et al. 1998) and H-Thr-2 (Yan et al. 2010) were used to amplify the Cytb gene fragment. The primers L3975 and H4551 (Lyu et al. 2021) were used to amplify the 16S gene fragment. The initial denaturation step was performed at 95 °C for 5 min; followed by 35 cycles of denaturation at 95 °C for 40 s, annealing at 53 °C for 40 s, and extension at 72 °C for 60 s; finally, extension was performed at 72 °C for 10 min. The PCR products were sequenced by General Biosystems (Anhui) Corp. Ltd. The sequencing results have been uploaded to GenBank, accession numbers are shown in Table 1. For phylogenetic analyses, we obtained the GenBank sequences of 14 Chinese *Japonigekko* species as ingroup, G. (Gekko) gecko and G. (Lomatodactylus) vittatus as outgroups. The detailed information of the sequences used in this study is shown in Table 1.

**Table 1.** DNA sequences, Localities, Voucher specimens, and GenBank accession numbers used in this study.

**No**.	**Species**	**Locality**	**Voucher**	**GenBank accession no**.		**Reference**
				**16S**	**Cytb**	
1	*Gekko luanchuanensis* sp. nov.	Luanchuan, Luoyang, Henan	HSR25010	PX831454	PX832420	This study
2	*G. luanchuanensis* sp. nov.	Luanchuan, Luoyang, Henan	HSR25011	PX831455	PX832421	This study
3	*G. luanchuanensis* sp. nov.	Luanchuan, Luoyang, Henan	HSR25012	PX831456	PX832422	This study
4	*G. luanchuanensis* sp. nov.	Boai, Henan	H8	—	GQ131344	Yan et al. (2010)
5	*G. luanchuanensis* sp. nov.	Luanchuan, Luoyang, Henan	H12	—	GQ131348	Yan et al. (2010)
6	*G. luanchuanensis* sp. nov.	Lintong, Shaanxi	H27	—	GQ131363	Yan et al. (2010)
7	*G. luanchuanensis* sp. nov.	Meixian, Shaanxi	H30	—	GQ131366	Yan et al. (2010)
8	*G. swinhonis*	Guzhen, Bengbu, Anhui	HSR25013	PX831457	PX832423	This study
9	*G. swinhonis*	Guzhen, Bengbu, Anhui	HSR25014	PX831458	PX832424	This study
10	*G. swinhonis*	Qingxu, Shanxi	H33	—	GQ131369	Yan et al. (2010)
11	*G. swinhonis*	Tianjin	H32	—	GQ131368	Yan et al. (2010)
12	*G. swinhonis*	Huaiyuan, Bengbu, Anhui	H24	—	GQ131360	Yan et al. (2010)
13	*G. swinhonis*	Hengshui, Hebei	H6	—	GQ131342	Yan et al. (2010)
14	*G. swinhonis*	Zunhua, Hebei	SYSr001814	MW451666	MW448296	Lyu et al. (2021)
15	*G. kaiyai*	Xinxian, Xinyang, Henan	AHUXXBH02	OQ780319	OQ743840	Zhang et al. (2023)
16	*G. kaiyai*	Shangcheng, Xinyang, Henan	AHUJGTBH01	OQ780322	OQ743843	Zhang et al. (2023)
17	*G. japonicus*	Zhoushan, Zhejiang	ZS01	–	GQ857616	Yan et al. (2010)
18	*G. japonicus*	Guanyang, Guangxi	SYSr001540	MW451656	MW448286	Lyu et al. (2021)
19	*G. japonicus*	Lushan, Jiangxi	SYSr001317	MW451649	MW448279	Lyu et al. (2021)
20	*G. auriverrucosus*	Yuncheng, Shanxi	YC01	–	GQ857615	Yan et al. (2010)
21	*G. adleri*	Daxin, Guangxi	SYSr001184	MW451636	MW448266	Lyu et al. (2021)
22	*G. adleri*	Ningming, Guangxi	SYSr001193	MW451640	MW448270	Lyu et al. (2021)
23	*G. cib*	Chengdu, Sichuan	CIB116961	MW451623	MW448256	Lyu et al. (2021)
24	*G. cib*	Chengdu, Sichuan	SYSr000708	MW451629	MW448260	Lyu et al. (2021)
25	*G. chinensis*	Neilingding Island, Guangdong	SYSr001157	MW451634	MW448264	Lyu et al. (2021)
26	*G. chinensis*	Shenzhen, Guangdong	SYSr001085	MW451632	MW448262	Lyu et al. (2021)
27	*G. hokouensis*	Mt. Wuy, Fujian	SYSr001290	MW451647	MW448277	Lyu et al. (2021)
28	*G. hokouensis*	Mt. Hengshan, Hunan	SYSr001319	MW451650	MW448280	Lyu et al. (2021)
29	*G. hokouensis*	Mt. Meiling, Jiangxi	SYSr001311	MW451648	MW448278	Lyu et al. (2021)
30	*G. kwangsiensis*	Wuming, Guangxi	SYSr001194	MW451641	MW448271	Lyu et al. (2021)
31	*G. melli*	Mt. Yinping, Guangdong	SYSr001146	MW451633	MW448263	Lyu et al. (2021)
32	*G. melli*	Mt. Yinping, Guangdong	SYSr001050	MW451631	MW448261	Lyu et al. (2021)
33	*G. palmatus*	Napo, Guangxi	SYSr001185	MW451637	MW448267	Lyu et al. (2021)
34	*G. similignum*	Mt. Wuzhi, Hainan	SYSr001597	MW451658	MW448288	Lyu et al. (2021)
35	*G. subpalmatus*	Zhoushan Island, Zhejiang	SYSr001381	MW451653	MW448283	Lyu et al. (2021)
36	*G. wenxianensis*	Wenxian, Gansu	WX1	—	EU417677	Zhou and Wang (2008)
**Outgroup**						
37	*G. gecko*	Nanning, Guangxi	N/A	AY282753	AY282753	Zhou et al. (2006)
38	*G. vittatus*	—	NUM-Az373	AB178897	AB178897	Kumazawa (2007)

The dataset was aligned using MEGA v. 11.0.11 with default parameters and aligned by MUSCLE (Edgar 2004; Tamura et al. 2021). The aligned datasets were concatenated in PhyloSuite v. 1.2.3. PartitionFinder2 was used to test the best partitioning scheme. Maternal phylogenetic reconstruction was performed using Bayesian-inference (BI) and maximum-likelihood (ML) methods based on the 16S and Cytb genes. BI analysis was performed in MrBayes v. 3.2.7a (Ronquist et al. 2012), and the ML analysis was conducted in IQ-TREE v. 2.2.3 (Minh et al. 2020). BI analysis was conducted in two independent runs, each comprising 10 million generations, with samples recorded every 1,000 generations and the first 25% discarded as burn-in. The analyses used the proportion of invariable sites estimated from the data and 1,000 bootstrap pseudoreplicates under the GTR+I+G model. The convergence of the MCMC chains was assessed using Tracer v. 1.7.2, with ESS for all parameters exceeding 200. Bayesian posterior probabilities (BPP) and ML bootstrap (BS) were labeled around the tree nodes. Uncorrected pairwise distance (*p*-distance) between inter- and intraspecies sequences was calculated using MEGA v. 11.0.11.

### Morphological examination

Morphological measurements, meristic characters, and their abbreviations follow Ma et al. (2024).

The following morphological characteristics were measured using digital calipers (model GB/T 21389–2008): (1) snout–vent length (SVL, from tip of snout to anterior margin of cloaca); (2) tail length (TaL, from posterior margin of cloaca to tip of tail); (3) axilla–groin length (AGD, distance between axilla and groin); (4) head length (HL, maximum head length from tip of snout to posterior margin of auricular opening); (5) head width (HW, maximum head width measured at the angle of the jaws); (6) head height (HH, maximum head height from the top of the head posterior to the eyes to the bottom of the lower jaw); (7) snout length (SL, from snout tip to anterior corner of eye); (8) eye–ear distance (EED: distance between posterior margin of eye to posterior margin of ear opening); (9) maximum eye diameter (ED); (10) forelimb length (FIL: length from the base of the palm to the elbow); (11) hindlimb length (HIL: distance from the base of heel to the knee). All measured traits, except for tail length (TaL) which was omitted due to a regenerative tail, were analyzed using ANCOVA with SVL as a covariate to test differences between *Gekko luanchuanensis* sp. nov. and *G. swinhonis*. Analyses were conducted in R v. 4.3.1 (Fox and Weisberg 2019, 2024).

The meristic features were taken as the following: (1) supralabials (SPL: number of scales from commissure of jaw to the rostral scale); (2) infralabials (IFL: number of scales from commissure of jaw to the mental scale); (3) interorbitals (IO: number of scales in a line between anterior corners of eyes); (4) postmentals (PM: scales bordering the mental); (5) dorsal tubercles row at midbody (DTR); (6) scales in a line from mental to the front of cloacal slit (SMC); (7) ventral scales at midbody from one ventrolateral fold to the other (V); (8) precloacal pores (PP); (9) preorbitals, number of scales in a line from the nostril to the anterior margin of the eye (PO); (10) subdigital lamellae under entire first finger (LF1); (11) subdigital lamellae under entire fourth finger (LF4); (12) subdigital lamellae under entire first toe (LT1); (13) subdigital lamellae under entire fourth toe (LT4); (14) postcloacal tubercles (PAT). Additionally, we documented the presence or absence of webbing (Web); and the presence of tubercles on the forelimbs (Fore tubercles), hindlimbs (Hind tubercles), and tail (Tail tubercles).

The data for the remaining species presented in Table 5 were compiled from the following references: Stejneger (1907); Zhou et al. (1982); Song (1985); Zhao et al. (1999); Goris and Maeda (2004); Rösler et al. (2005); Toda et al. (2008); Zhou and Wang (2008); Rösler et al. (2010); Phung and Ziegler (2011); Nguyen et al. (2013); Luu et al. (2014, 2015, 2017); Ngo et al. (2015); Yang (2015); Hou et al. (2021); Lyu et al. (2021); Sitthivong et al. (2021); Zhang et al. (2023); Ma et al. (2024); Wang et al. (2024); Xu et al. (2025); Wang et al. (2026).

## Results

Morphologically, this new species can be distinguished from all known congeners (details are provided in the taxonomic account below). Morphometric data were analyzed for specimens of *G. luanchuanensis* sp. nov. and its sister species *G. swinhonis* from Anhui. After accounting for the effect of SVL, *G. luanchuanensis* sp. nov. differs significantly from *G. swinhonis* in HL, FIL (R), and HIL (*P* < 0.05; Table 4).

**Figure 2.**
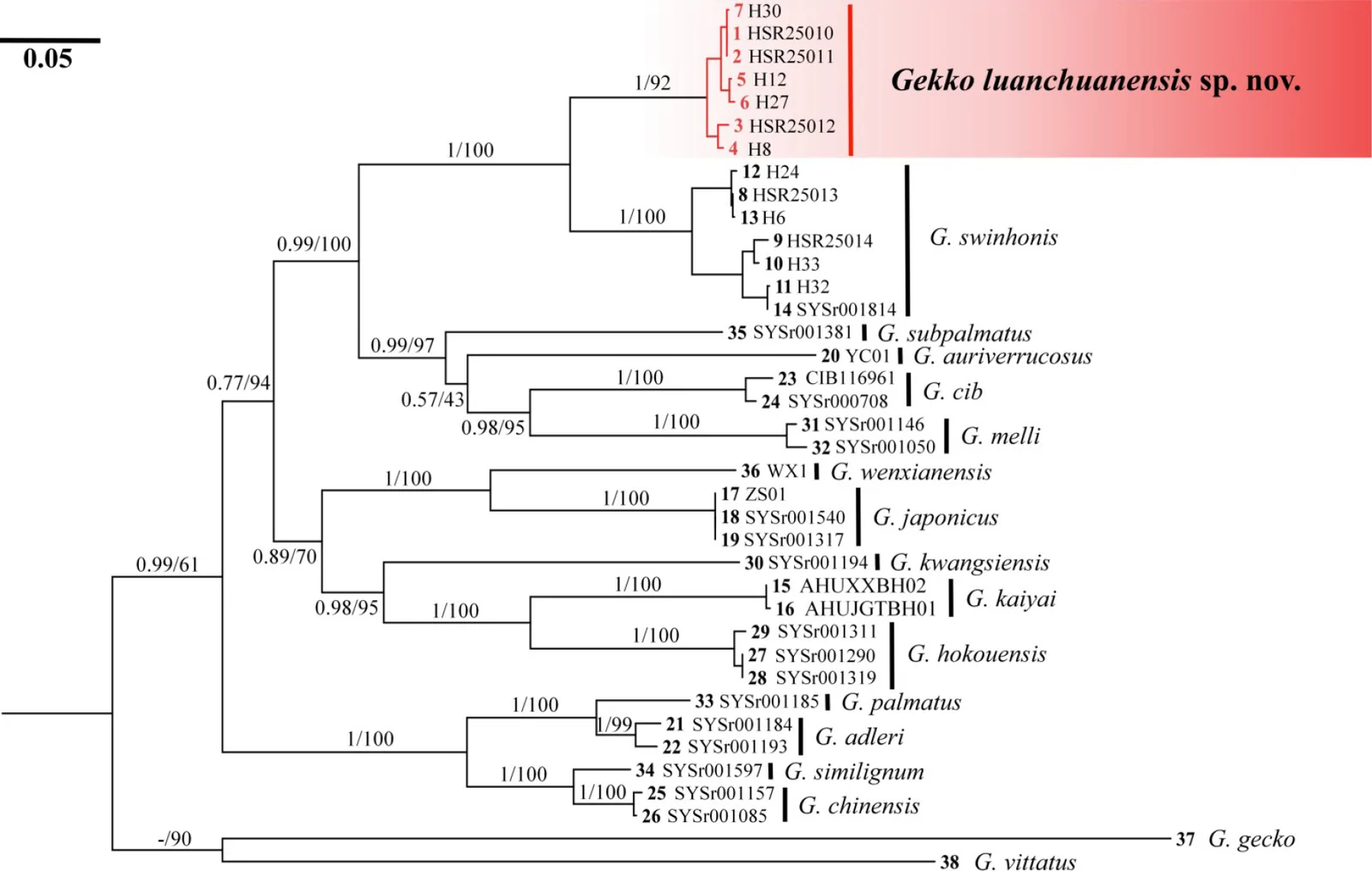
Maximum-likelihood (ML) tree topology inferred from concatenated 16S and Cytb gene fragments (1636 bp). The Bayesian-inference (BI) tree exhibits a topology similar to that recovered by the ML analysis. Numbers before the diagonal indicate Bayesian posterior probability (BPP), and those after the diagonal indicate ML bootstrap (BS). *Gekko luanchuanensis* sp. nov. are shown in red. Numbers at branch tips correspond to No. and Voucher in Table 1.

The concatenated dataset in this study comprises 1636 bp, including 556 bp of the 16S rRNA gene and 1080 bp of the Cytb gene. Both BI and ML analyses yielded similar topological structures (Fig. 2). This new species forms a strongly supported independent clade (BPP = 1.00 and BS = 92), with *G. swinhonis* recovered as its closest relative. The uncorrected *p*-distances among some species of the subgenus *Japonigekko* were calculated based on mitochondrial 16S and Cytb gene fragments. The genetic distances between *G. luanchuanensis* sp. nov. and its congeners range from 4.86% (vs *G. swinhonis*) to 16.47% (vs *G. palmatus*) for 16S, and from 12.22% (vs *G. swinhonis*) to 21.67% (vs *G. kaiyai*) for Cytb, indicating distinct interspecific genetic differentiation between *G. luanchuanensis* sp. nov. and other recognized congeners. The uncorrected group mean *p*-distance, based on concatenated 16S and Cytb gene fragments used in this study, is presented in Table 2.

**Table 2.** Uncorrected *p*-distance (%) of some Gekko (Japonigekko) species based on partial mitochondrial 16S and Cytb gene sequences. Numbers refer to specimens listed in Table 1. (Bold values represent Estimates of Average Evolutionary Divergence over Sequence Pairs within Groups).

**Species**	**1**	**2**	**3**	**4**	**5**	**6**	**7**	**8**	**9**	**10**	**11**	**12**	**13**	**14**	**15**
1. *G. luanchuanensis* sp. nov.	**1.61**														
2. *G. swinhonis*	13.99	**4.43**													
3. *G. kaiyai*	20.80	21.33	**0.28**												
4. *G. japonicus*	21.07	20.32	19.92	**0**											
5. *G. auriverrucosus*	18.22	18.16	20.06	21.05	—										
6. *G. adleri*	19.96	22.40	19.70	20.41	22.25	**2.82**									
7. *G. cib*	19.72	19.72	22.03	20.69	19.07	19.10	**1.98**								
8. *G. chinensis*	20.34	20.86	21.33	19.63	18.57	14.41	20.27	**0.56**							
9. *G. hokouensis*	20.09	21.62	15.58	19.07	20.24	19.68	22.88	20.06	**0.75**						
10. *G. kwangsiensis*	18.12	18.50	18.50	20.62	20.20	20.34	20.83	19.92	17.66	—					
11. *G. melli*	19.75	20.77	21.47	21.47	18.64	21.15	17.58	18.71	21.59	19.28	**1.84**				
12. *G. palmatus*	19.98	22.38	20.62	21.61	22.32	7.13	20.90	15.96	20.24	20.06	21.26	—			
13. *G. similignum*	19.49	22.42	20.90	20.20	20.06	14.34	19.84	6.07	20.34	19.63	19.70	14.97	—		
14. *G. subpalmatus*	17.84	17.05	21.19	19.77	16.67	21.54	17.37	19.77	21.00	18.22	17.37	22.03	20.62	—	
15. *G. wenxianensis*	18.79	19.27	22.03	14.83	19.21	23.38	21.33	21.19	20.20	20.48	21.96	22.74	21.33	19.63	—

Phylogenetic analyses demonstrated that the gecko population from Luanchuan County, Henan Province, China, forms a distinct and strongly supported monophyletic lineage with the subgenus *Japonigekko*. Combined with morphological evidence, these results support the recognition of this gecko population as a new species.

## Taxonomic account

**Family Gekkonidae Oppel, 1811**

**Genus *Gekko* Laurenti, 1768**

**Subgenus *Japonigekko* Wood, Guo, Travers, Su, Olson, Bauer, Grismer, Siler, Moyle, Andersen & Brown, 2020**

### Gekko luanchuanensis

Taxon classificationAnimaliaSquamataGekkonidae

Zhang, Jiang, Huang & Wu sp. nov.

F9494803-7626-5F90-864B-8A8EA3783077

https://zoobank.org/B0397937-1623-4B35-977E-54A843563156

#### Type material.

• ***Holotype*** HSR25011, adult male, from County Luanchuan, Henan Province, China (33°78'N, 111°61'E; 782 m asl.), Yixue Zhang leg., 22 August 2025.

• ***Paratypes*** (*n* = 2, all from Henan Province, China), 2 paratype specimens, subadult female (HSR25010), juvenile female (HSR25012), same data as holotype, except 22–23 August 2025.

#### Type localities of congeners.

*Gekko swinhonis*, Pekin (Beijing), China (Boulenger 1885); *G. japonicus*, Japan; *G. kaiyai*, Liankang Mountain National Nature Reserve, Xinyang City, Henan Province, China; *G. auriverrucosus*, Hejin (Hojin) Prefecture, Shanxi Province, China; *G. adleri*, karst forest near Ban Coong Village, in Duc Quang Commune, Ha Lang District, Cao Bang Province, northern Vietnam; *G. cib*, wall surface in the Herpetological Museum, Chengdu Institute of Biology, Chinese Academy of Sciences, Chengdu City, Sichuan Province, China; *G. chinensis*, China (probably Hongkong); *G. hokouensis*, Yanshan City, Jiangxi Province, China; *G. kwangsiensis*, Wuming County, Nanning City, Guangxi Zhuang Autonomous Region, China; *G. melli*, northeastern Guangdung Province, China; *G. palmatus*, Man-son Mountains, Tonking, northern Vietnam; *G. similignum*, Five Finger Mountain, Anmao City, Hainan Province, China; *G. subpalmatus*, China (probably Honkong); *G. wenxianensis*, Danbao Village on the south slope of the west Qinling Mts, Wenxian County, Gansu Province, China; *G. fengshanensis*, Fenghuang Village, Fengcheng Town, Fengshan County, Hechi City, Guangxi Zhuang Autonomous Region, China; *G. alpinus*, Zhubalong Village, Batang County, Ganzi Zang Autonomous Prefecture, Sichuan Province, China; *G. jinjiangensis*, Benzilan Town, Deqin County, Yunnan Province, China; *G. paucituberculatus*, Tianyang District, Baise City, Guangxi Zhuang Autonomous Region, China; *G. liboensis*, Chengguan (Chengkwang), Libo Prefecture, Guizhou, southern China; *G. scabridus*, Yongren (Yongzen) Prefecture, Yunnan Province, China; *G. taibaiensis*, Taibai County, Shaanxi Province, China; *G. tesselatus*, Se’ergu Town, Heishui County, Aba Tibetan and Qiang Autonomous Prefecture, Sichuan Province, China; *G. ichangensis*, Muqiao Creek Village, Gaojiayan Town, Changyang Tujia Autonomous County, Yichang City, Hubei Province, China; *G. prep*, Hainan Province, China; *G. guishanicus*, Guishan Isle, Toucheng Township, Yilan County, northeast Taiwan, China; *G. liui*, Dazhou Island, Wanning City, Hainan Province, China; *G. mosaicus*, Longtan Town, Yuanjiang Hani, Yi and Dai Autonomous County, Yunnan Province, China (Uetz et al. 2026).

#### Etymology.

The specific name is a Latinized adjective meaning “originating from Luanchuan”, which refers to the county where the type locality is located. For common names, we recommend using “Luanchuan Gecko” (English) and “栾川壁虎 (luán chuān bì hǔ)” (Chinese).

#### Diagnosis.

*Gekko luanchuanensis* sp. nov. can be distinguished from other *Japonigekko* species by the following combination of characteristics: (1) body size moderate (SVL of 46.52 mm in adult males); (2) nares connected to the rostrals; (3) interorbital 23–25; (4) tubercles small and sparse, and dorsal tubercles not in rows at midbody; (5) 141–157 scales in a line from mental to the front of cloacal slit; (6) 35–37 ventral scale rows; (7) limbs without tubercles; (8) webbing absent; (9) subdigital lamellae 9–11 on finger I, 10–14 on finger IV, 9–10 on toe I, and 9–13 on toe IV; (10) 6–8 precloacal pores; (11) two postcloacal tubercles on each side; (12) 6–8 dark-gray stripes on back extending from neck to tail base.

#### Description of the holotype

**Figure 3.**
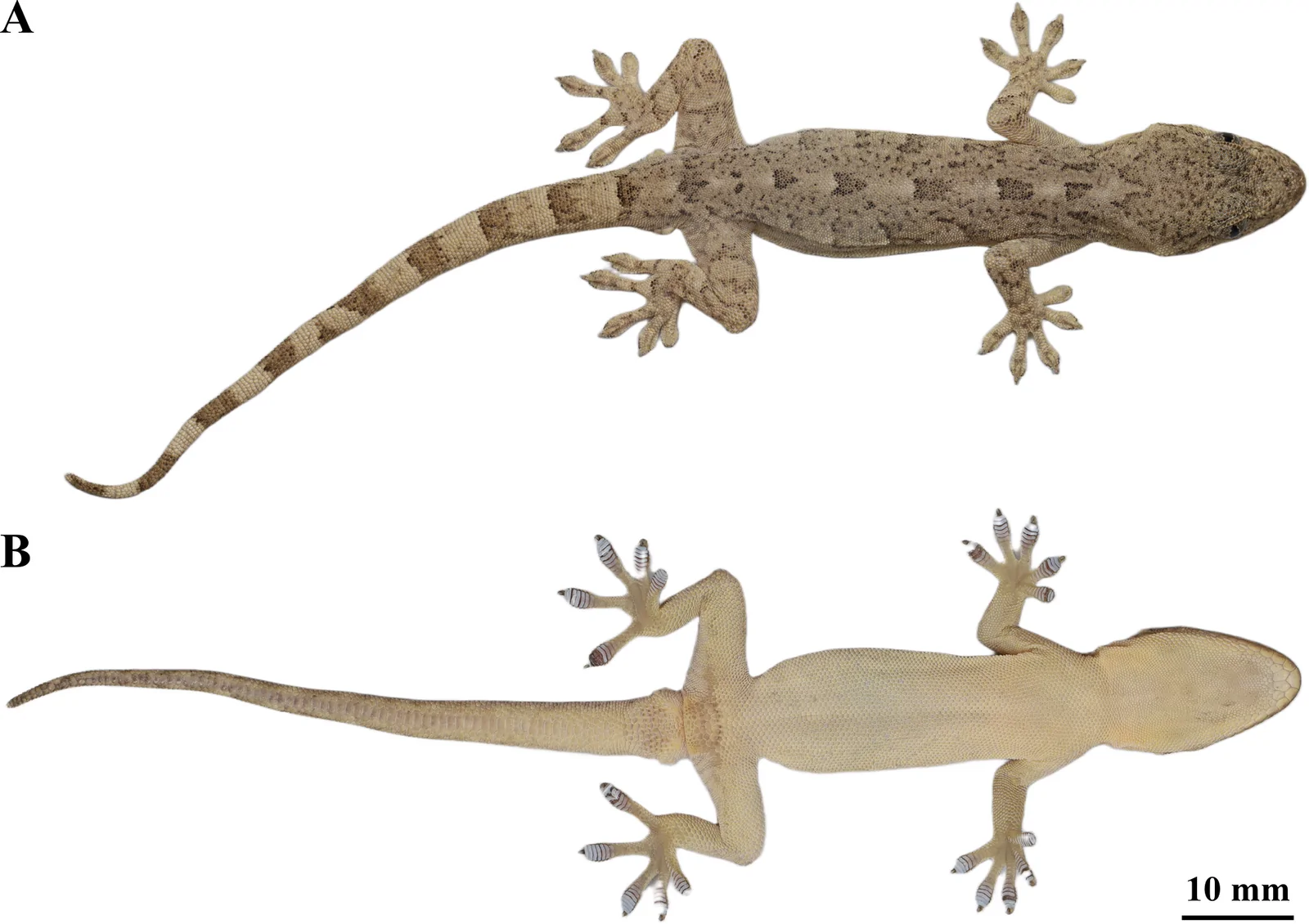
Holotype (HSR25011, adult male) of *Gekko luanchuanensis* sp. nov. **A**. Dorsal view of body; **B**. Ventral view of body. Photographs by YXZ.

**Figure 4.**
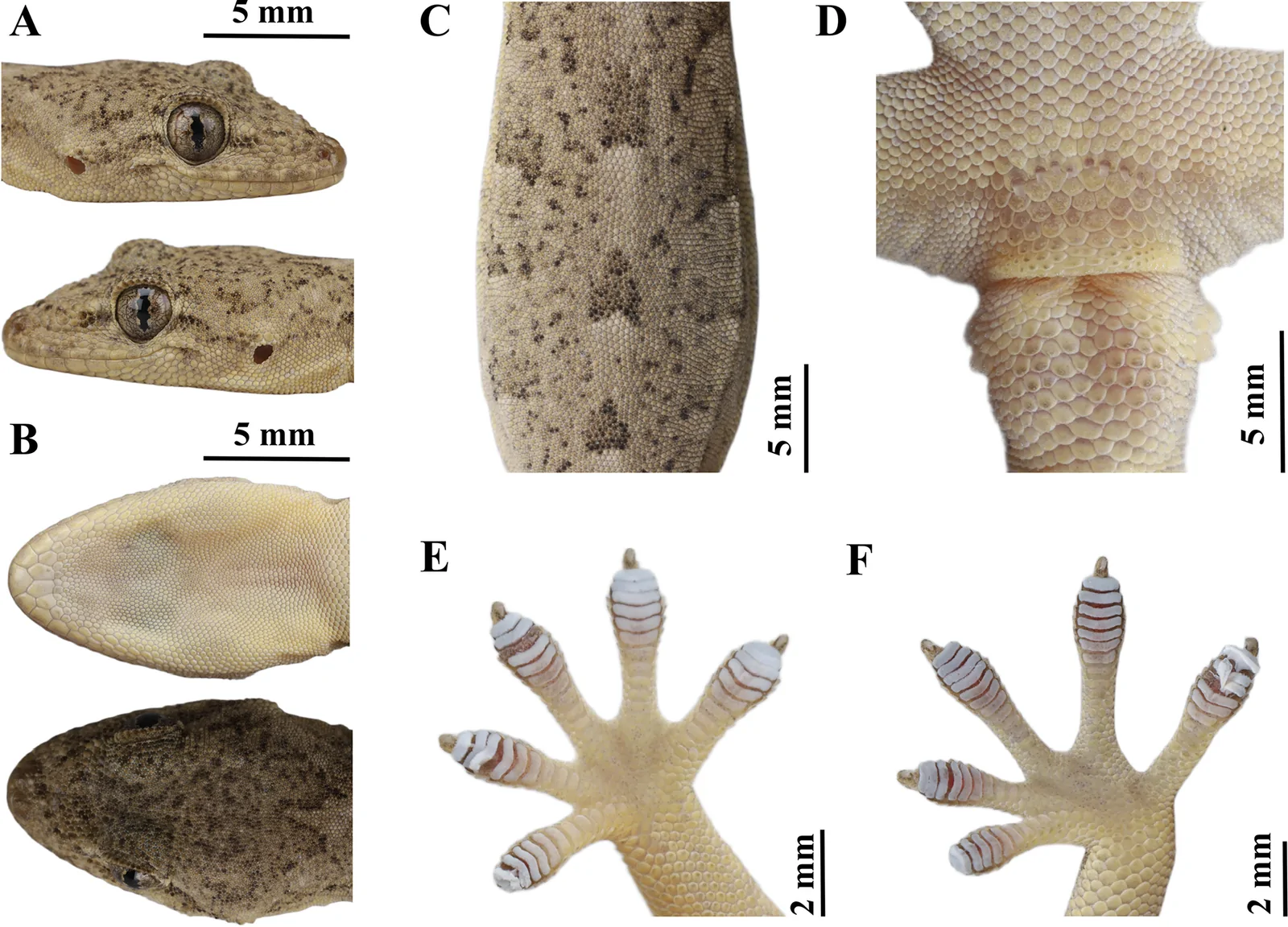
Holotype (HSR25011, adult male) of *Gekko luanchuanensis* sp. nov. **A**. Right and left lateral view of head; **B**. Dorsal and ventral view of head; **C**. Dorsal view of middle body; **D**. Ventral view of precloacal region; **E**. Ventral view of left hand; **F**. Ventral view of left foot. Photographs by YXZ.

**(Figs 3, 4)**. Adult male, medium-sized, SVL 46.52 mm; Tal 54.79 mm; head length greater than head width, HL/HW ratio 1.246; neck distinct; rostral scale enlarged, pentagonal; nostril elliptical, in contact with the rostral and the first supralabial; supralabials 10/11 (left/right); preoculars 14/15 (left/right); interorbitals 23; head scales gradually decreasing in size from snout to neck; dorsal head without tubercles; pupil vertical, featuring crenulated edges; ear opening elliptical; mental pentagonal, wider than long; two postmentals, hexagonal, longer than wide, in contact with first infralabial; infralabials 8/8 (left/right); seventh infralabial on the left side formed by incomplete fusion of three scales, and the eighth infralabial on the right side formed by incomplete fusion of two scales, both counted as a single scale.

Body flattened; dorsal scales round, granular, tightly packed; dorsal tubercles very few, nearly elliptical convexities, slightly larger than dorsal scales, no rows of tubercles on back; ventrolateral fold distinct, without tubercles; belly milky yellow; 153 ventral midline scales; ventrals relatively large, fish-scale-like, imbricate, gradually becoming smaller and round or hexagonal in the gular region; precloacal scales enlarged, precloacal pores 8, arranged in an arc.

Limbs slender, without tubercles; granular scales on limbs slightly larger than those on dorsal, round and granular, juxtaposed; scales on palms and soles to digits transitioning from round, granular, and juxtaposed to flattened and imbricate distally; tips of digits dilated, claws absent on the first finger and first toe, present on remaining digits; webbing absent. Subdigital lamellae undivided, left manus 10–9–11–11–10, right manus 10–9–11–11–10; left pes 10–9–11–9–10, right pes 9–10–10–9–9. Relative finger length: IV > III > V > II > I; relative toe length: IV > III > V > II > I.

Tail base distinctly swollen; postcloacal tubercles 2/2, large, conical; tail gradually tapering from the swollen base, elliptical in cross-section. Dorsal caudal scales granular, slightly larger than dorsal scales, arranged in annular rows; tubercles absent on tail. Ventral scales at tail base granular, fan-shaped, imbricate, transitioning posteriorly into enlarged, smooth, imbricate subcaudal plates.

#### Coloration of the holotype in life

**(Figs 3, 4)**. In life, dorsal ground color of head and body light brown; dorsal head with dense, dark-brown markings; posterior portion of snout with a dark-brown, transverse streak across it, extending from mid-level between anterior margin of orbit and nostril, passing through eye, and connecting to anterior margin of ear opening; upper labial margin with light-brown suffused blotches; corner of mouth with a few brown spots. Vertical pupil black, featuring crenulated edges; iris yellowish brown, with reticulated pattern. Junction of head and neck with a W-shaped dark-brown marking; dorsum with 7 grayish-brown stripes along vertebral line from neck to tail base, posterior margins dark gray; irregular grayish-brown blotches on both sides of dorsum, interspersed with a few small black spots; limbs with grayish-brown stripes. Tail with 10 brownish-yellow rings from base, margins dark gray, rings gradually widening toward tail tip. Ventral surface of body yellowish white; ventral surface of tail with black spots appearing posteriorly from mid-section.

#### Comparisons.

To minimize the influence of age on morphometric data, juveniles were excluded from the morphological analyses (Wotherspoon and Burgin 2011). Morphological data are presented in Table 3.

**Table 3.** The morphological data (unit: mm) and scale characteristics of *Gekko luanchuanensis* sp. nov. are presented, with * indicating regenerative tail. Bilateral morphological measurements and meristic features are expressed as left/right. The meanings of abbreviations are detailed in the Material and Methods section.

**Voucher Number**	**HSR25011**	**HSR25010 (Subadult)**	**HSR25012 (juvenile)**
**Type**	Holotype	Paratypes	Paratypes
**Sex**	Male	Female	Female
**SVL**	46.52	33.68	28.67
**TAL**	54.79	27.06*	30.34
**AGD**	20.07	13.87	11.86
**HL**	11.66	9.23	7.77
**HW**	9.36	7.90	6.99
**HH**	5.02	3.83	3.36
**SL**	4.06	3.91	3.05
**EED**	3.93	3.18	3.02
**ED**	2.25	2.16	2.15
**FIL**	6.35/6.31	5.01/4.92	4.02/4.11
**HIL**	8.20/8.01	5.75/5.64	5.25/5.05
**SPL**	10/11	12/11	13/12
**IFL**	8/8	10/9	11/11
**IO**	25	23	23
**PM**	2	2	2
**DTR**	0	0	0
**SMC**	153	141	157
**V**	35	37	37
**LF1**	10/10	9/9	9/11
**LF4**	11/11	14/12	10/10
**LT1**	10/9	10/10	10/10
**LT4**	9/9	10/11	10/13
**PP**	8	6	–
**PAT**	2/2	2/2	2/2
**PO**	14/15	13/14	14/15

**Figure 5.**
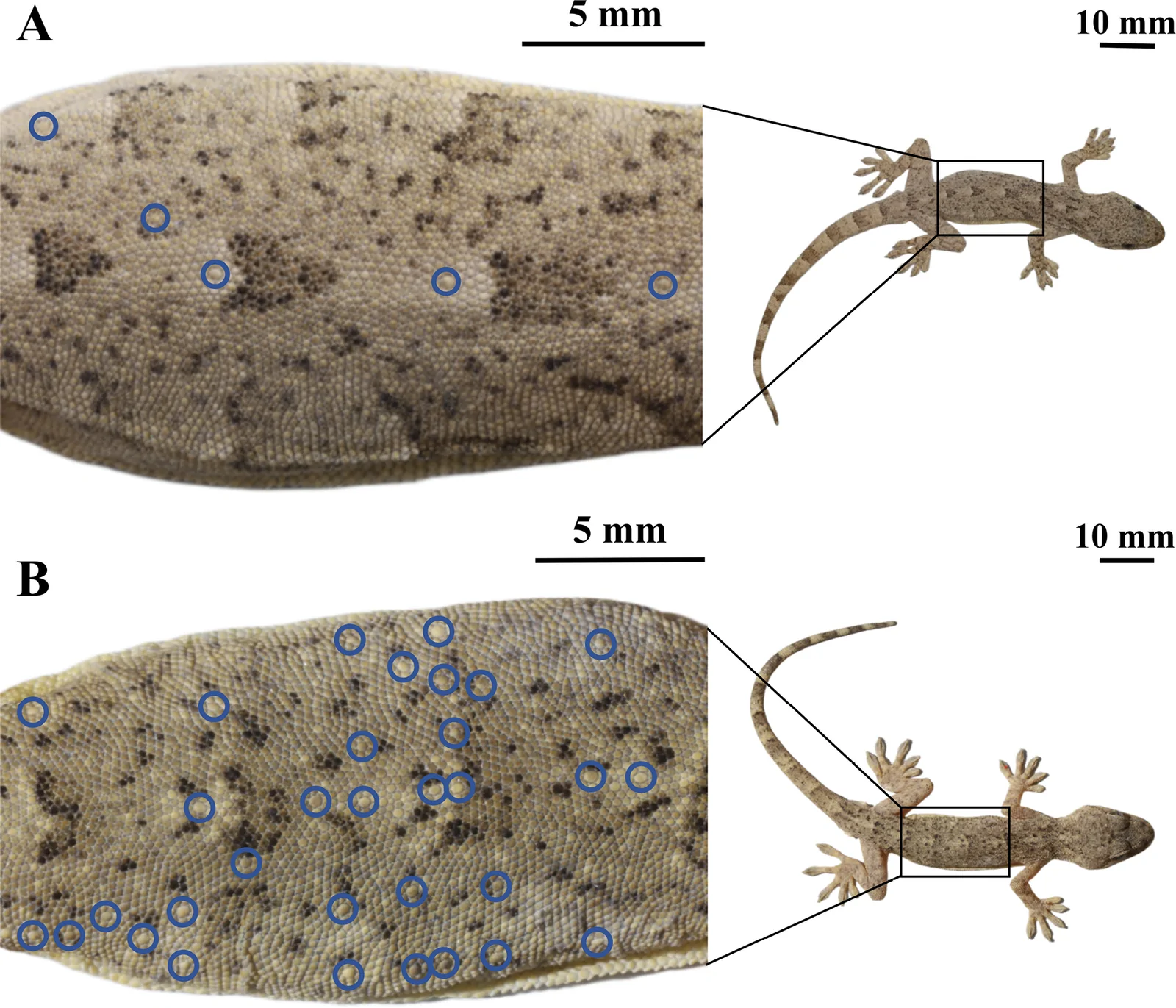
Comparison of the number of verrucous granules on the dorsal surfaces of *Gekko luanchuanensis* sp. nov. (**A**) and *G. swinhonis* (**B**). Tubercles are circled in blue. Photographs by YXZ.

The morphological data of *G. luanchuanensis* sp. nov. were compared with *G. swinhonis*, and they can be distinguished by the following characteristics (1) smoother body surface with fewer tubercles, and dorsal tubercles not in rows at midbody (Fig. 5) (vs *G. swinhonis* has more tubercles on the body surface, with 6–9 rows of tubercles on the dorsal); (2) 23–25 interorbitals (vs 20–23); (3) relative length of toes: IV > III > V > II > I (vs IV > V > III > II > I). The results of the morphometric ANCOVA are shown in Table 4, indicating significant differences between *G. luanchuanensis* sp. nov. from Luanchuan County and *G. swinhonis* from Anhui Province in HL, FIL (R), and HIL (L/R).

**Table 4.** Test for differences between *Gekko luanchuanensis* sp. nov. and *G. swinhonis* using ANCOVA (SVL as covariate). * *P* < 0.05.

**Species**	**Holotype (HSR25011)**	***G. luanchuanensis* sp. nov. (*n* = 2)**	***G. swinhonis* (*n* = 4)**	***F***	***P***
**AGD**	20.07	13.87–20.07 (16.97±3.1)	18.21–23.91 (21.06±2.85)	7.898	0.114
**HL**	11.66	9.23–11.66 (10.45±1.22)	11.57–12.46 (12.02±0.45)	199.841	0.005*
**HW**	9.36	7.90–9.36 (8.63±1.63)	9.16–10.56 (9.86±0.7)	16.102	0.059
**HH**	5.02	3.83–5.02 (4.43±0.6)	4.78–5.33 (5.06±0.28)	18.956	0.051
**SL**	4.06	3.91–4.06 (3.99±0.08)	5.36–6.06 (5.71±0.35)	13.885	0.068
**EED**	3.93	3.18–3.93 (3.56±0.38)	4.00–5.29 (4.65±0.65)	6.774	0.131
**ED**	2.25	2.16–2.25 (2.21±0.05)	2.42–2.92 (2.67±0.25)	1.422	0.438
**FIL(L)**	6.35	5.01–6.35 (5.68±0.67)	6.14–6.92 (6.53±0.39)	13.109	0.072
**FIL(R)**	6.31	4.92–6.31 (5.62±0.7)	6.15–6.92 (6.54±0.39)	38.751	0.025*
**HIL(L)**	8.20	5.75–8.20 (6.98±1.23)	7.21–8.60 (7.91±0.7)	58.045	0.017*
**HIL(R)**	8.01	5.64–8.01 (6.83±1.19)	7.36–8.83 (8.10±0.74)	21.451	0.045*

Phylogenetic analyses recovered *G. luanchuanensis* sp. nov. in close association with *G. auriverrucosus*, *G. cib*, *G. melli*, and *G. subpalmatus*. However, the new species can be distinguished from these species by several morphological characters.

*Gekko luanchuanensis* sp. nov. can be distinguished from *G. auriverrucosus* by (1) interorbitals (23–25 vs 26–27); (2) dorsal tubercles (0 vs 16–20); (3) subdigital lamellae under entire first finger (9–10 vs 6–8); (4) subdigital lamellae under entire fourth finger (9–13 vs 6–8); (5) no tubercles present on limbs and tail.

*Gekko luanchuanensis* sp. nov. can be distinguished from *G. cib* by (1) interorbitals (23–25 vs 28–36); (2) scales in a line from mental to the front of cloacal slit (141–157 vs 171–196); (3) webbing absent.

*Gekko luanchuanensis* sp. nov. can be distinguished from *G. melli* by (1) smaller body size; (2) interorbitals (23–25 vs 34–40); (3) scales in a line from mental to the front of cloacal slit (141–157 vs 171–192); (4) ventral scales at midbody from one ventrolateral fold to the other (35–37 vs 44–46); (5) webbing absent; (6) precloacal pores (6–8 vs 9–11).

*Gekko luanchuanensis* sp. nov. can be distinguished from *G. subpalmatus* by (1) interorbitals (23–25 vs 28–37); (2) ventral scales at midbody from one ventrolateral fold to the other (35–37 vs 39–46); (3) webbing absent.

This species can be distinctly distinguished from other species of *Japonigekko* through a series of unique characteristics. The differences between the new species and other *Japonigekko* members distributed in China are shown in Table 5.

**Table 5.** Comparison of morphological characteristics of *Japonigekko* species in China. “—” denotes unavailable data; bold indicates differences from new species. Data of *G. swinhonis* are from this study (*n* = 4). “1” and “0” represent presence or absence.

**No**.	**Species**	**MaxSVL**	**SPL**	**IFL**	**IO**	**DTR**	**SMC**	**V**	**LT1**	**LT4**	**Web**	**Fore tubercles**	**Hind tubercles**	**Tail tubercles**	**PP**
1	*Gekko luanchuanensis* sp. nov.	46.52	10–13	8–11	23–25	0	141–157	35–37	9–10	9–13	0	0	0	0	6–8
2	*G. swinhonis*	49.45	10–12	8–10	**20–23**	**6–9**	148–165	34–39	9–11	10–14	0	0	0	0	7–9
3	*G. kaiyai*	**64.99**	9–12	9–13	22–33	**11–18**	157–209	30–43	8–9	7–11	0	**1**	**1**	**1**	**9–12**
4	*G. japonicus*	**74**	9–13	8–13	**32–35**	**9–14**	**169–188**	39–44	10–12	**14–16**	0	**1**	**1**	**1**	4–9
5	*G. auriverrucosus*	**69**	9–11	9–11	**26–27**	**16–20**	—	—	**6–8**	**6–8**	0	**1**	**1**	**1**	8–11
6	*G. adleri*	**75.3**	10–15	9–13	**27–36**	**7–11**	**168–190**	35–44	**11–14**	11–15	**1**	0	**1**	**1**	**17–21**
7	*G. cib*	**66.4**	10–12	10–14	**28–36**	0	**171–196**	37–45	9–13	9–17	**1**	0	0	0	7–9
8	*G. chinensis*	**72**	10–14	9–13	**35–48**	**10**	156–167	37–39	8–10	9–12	**1**	0	**1**	**1**	**17**–**27**
9	*G. hokouensis*	**70**	10–14	8–11	**30–33**	**12–18**	153–174	36–43	8–11	**15–18**	0	0	0	**1**	5–9
10	*G. kwangsiensis*	**69.7**	10–12	11–13	**29–31**	**9–11**	**185–208**	**41–45**	**11–13**	13–18	**1**	0	0	**1**	**9**–**11**
11	*G. melli*	**80.3**	10–13	9–12	**34–40**	0	**171–192**	**44–46**	10–12	11–14	**1**	0	0	0	**9**–**11**
12	*G. palmatus*	**79.7**	11–15	9–13	**27–36**	**4–12**	**160–191**	36–47	10–13	10–16	**1**	0	0	**1**	**23**–**30**
13	*G. similignum*	58.9	12–14	11	**46–48**	**11**	—	—	**11–13**	12–14	**1**	0	0	**1**	**17**
14	*G. subpalmatus*	**65.8**	8–12	7–12	**28–37**	0	144–190	**39**–**46**	9–12	11–14	**1**	0	0	0	5–9
15	*G. wenxianensis*	59	12	11	—	**10**	—	**42**–**44**	**6**	9	0	0	**1**	—	6–8
16	*G. alpinus*	**74.16**	9–13	8–10	22–28	**12–17**	158–189	32–39	8–11	13–15	0	**1**	**1**	**1**	4–7
17	*G. fengshanensis*	**79.9**	9–13	10–13	22–27	**9–11**	**197–213**	**40**–**49**	**12–14**	13–15	**1**	0	0	0	**9**–**12**
18	*G. jinjiangensis*	61.6	7–10	6–9	20–24	**12–16**	146–169	31–47	8–11	11–15	0	**1**	**1**	**1**	**4–5**
19	*G. liboensis*	**85**	12	11	**40**	**10**	—	—	**8**	9	0	0	0	—	—
20	*G. paucituberculatus*	**85.9**	11	9–10	**37**	**4**	**189–192**	**42–44**	**11**	11–13	0	0	0	0	**12**
21	*G. scabridus*	64	9–11	9–11	**30**	**17**–**21**	—	—	6–9	7–9	0	**1**	**1**	**1**	**10–15**
22	*G. taibaiensis*	**69**	9–10	8–10	**28**	—	—	—	**6**–**7**	**7–8**	—	—	—	—	4–6
23	*G. tesselatus*	**65.3**	10–11	9–12	22–26	**12–15**	130–157	31–39	7–9	9–12	0	0	**1**	**1**	6–8
24	*G. ichangensis*	**66.5**	7–11	7–11	27–36	**10–12**	**174–195**	**39–47**	**12–15**	13–18	**1**	**1**	**1**	**1**	5–8
25	*G. prep*	**75.7**	**14**	11	37–38	**12**	144–152	**38–40**	**11–12**	12–13	**1**	0	0	**1**	**24**
26	*G. guishanicus*	**64**	—	—	—	0	—	—	8–10	8–10	**1**	0	0	0	6–8
27	*G. liui*	56.67	11–13	11–12	**37–42**	**7–8**	140–148	34–39	10–11	12–13	**1**	0	0	**1**	**20–23**
28	*G. mosaicus*	**65.3**	11	9–10	24–26	**15–19**	135–142	**27–32**	9–11	10–12	0	**1**	**1**	**1**	**17–19**

*Gekko luanchuanensis* sp. nov. can be distinguished from *G. japonicus*, *G. adleri*, *G. chinensis*, *G. hokouensis*, *G. kwangsiensis*, *G. palmatus*, *G. similignum*, *G. liboensis*, *G. paucituberculatus*, *G. scabridus*, *G. taibaiensis* and *G. liui* by interorbitals 23–25.

*Gekko luanchuanensis* sp. nov. can be distinguished from *G. kaiyai*, *G. wenxianensis*, *G. alpinus*, *G. jinjiangensis*, *G. fengshanensis*, *G. tesselatus*, *G. ichangensis*, *G. prep* and *G. mosaicus* by dorsal tubercle not rows at midbody.

*Gekko luanchuanensis* sp. nov. can be distinguished from *G. guishanicus* by webbing absent.

#### Distribution and habitat

**Figure 6.**
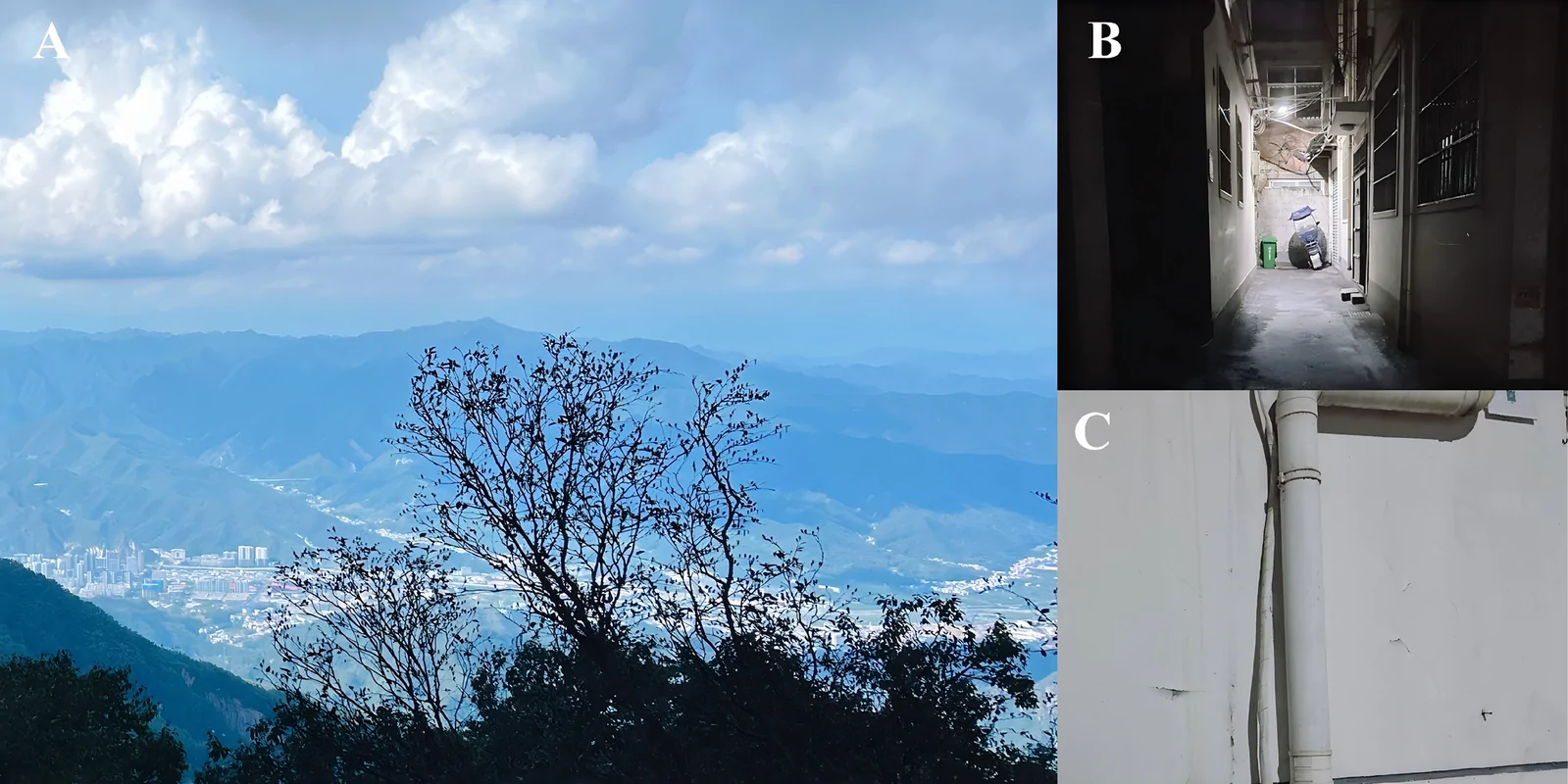
Habitat of *Gekko luanchuanensis* sp. nov. **A**. Luanchuan County, Henan Province; **B**. Habitat of holotype HSR25011; **C**. Microhabitat. Photographs by YXZ.

**(Fig. 6)**. *Gekko luanchuanensis* sp. nov. is currently known only from the Qinling Mountains in Henan and Shaanxi Provinces, and the Taihang Mountains in Shanxi Province. All samples used in this study were collected at night during August and September, with an average nighttime temperature of 23–25 °C and at altitudes of 754–782 m. Its habitat primarily consists of walls and crevices of artificial structures.

#### Additional specimens examined.

*Gekko swinhonis* (*n* = 4): HSR25013 (adult male), HSR25014 (adult female), HSR25015 (adult female), HSR25016 (adult female), Guzhen County, Bengbu City, Anhui Province, China, Yixue Zhang leg., 25 August 2025.

The corresponding morphological data of the above samples are shown in Tables 4, 5, and the GenBank accession numbers of the HSR25013 and HSR25014 specimens are listed in Table 1. These specimens are preserved at Anhui Normal University in China.

## Discussion

This study employed an integrated taxonomic approach combining molecular phylogenetic and morphological evidence to support the recognition of *Gekko luanchuanensis* sp. nov. as a new species distinct from others in the genus *Gekko*. The discovery of this new species increases the number of recognized *Gekko* species worldwide to 102. Of these, 40 species belong to the subgenus *Japonigekko*, with 28 of these occurring in China.

Phylogenetic analyses revealed that *G. luanchuanensis* sp. nov. and *G. swinhonis* form a well-supported monophyletic clade (BPP = 1, BS = 100), indicating their shared evolutionary origin. It has been hypothesized that the divergence between these two gecko species was associated with the Qinling orogeny (Yan et al. 2010).

The Qinling Mountains were originally formed through the collision of the North and South China Blocks during the Paleozoic and Triassic periods. These mountains form a natural north–south boundary between the Palaearctic and Oriental realms in China. The unique geographical location and diverse climate types make the Qinling Mountains a unique area in terms of biodiversity in the world (Dong et al. 2022). The discovery of *G. luanchuanensis* sp. nov. enriches the species diversity of the Qinling Mountains.

Currently, *G. luanchuanensis* sp. nov. is found in the Qinling Mountains in Henan and Shaanxi Provinces and the Taihang Mountain in Shanxi Province, while *G. swinhonis* is widely distributed across most of the plains in northern China. We hypothesize that the other areas surrounding the Qinling and Taihang Mountains may also provide suitable habitats for this new species.

## Supplementary Material

XML Treatment for Gekko luanchuanensis

## Additional information

### Artificial Intelligence (AI) use

The authors accept full responsibility for the content of the manuscript, including the disclosure of any use of AI.

No AI tools were used in the preparation of this manuscript.

### Author ORCIDs

Y. Zhang https://orcid.org/0009-0009-3763-3649

Z. Jiang https://orcid.org/0009-0004-1492-0665

R. Huang https://orcid.org/0000-0002-1004-3835

### Data availability

All of the data that support the findings of this study are available in the main text.
